# Targeted Therapies for Epstein-Barr Virus-Associated Lymphomas

**DOI:** 10.3390/cancers12092565

**Published:** 2020-09-09

**Authors:** Yonggang Pei, Josiah H. Y. Wong, Erle S. Robertson

**Affiliations:** Department of Otorhinolaryngology-Head and Neck Surgery, and Microbiology, Tumor Virology Program, Abramson Comprehensive Cancer Center, Perelman School of Medicine at the University of Pennsylvania, Philadelphia, PA 19104, USA; peiy@pennmedicine.upenn.edu (Y.P.); Josiah.Wong@pennmedicine.upenn.edu (J.H.Y.W.)

**Keywords:** targeted therapies, Epstein-Barr virus, lymphomas

## Abstract

**Simple Summary:**

Epstein-Barr virus (EBV) is the first-discovered and important human tumor virus. It infects more than 90% of human population and induces various lymphomas. Development of specific targeted therapies is very critical for treatment of EBV-induced lymphomas, but it remains a great challenge. In this review, we introduced the current progress of EBV-specific therapies and the promising approaches that can be developed as novel targeted therapies, which involve protective or therapeutic strategies to target these lymphomas on different levels. This work will provide new insights into the development of new targeted therapies against EBV-associated lymphomas.

**Abstract:**

The Epstein-Barr virus (EBV) is the first human tumor virus identified that can transform quiescent B lymphocytes into lymphoblastoid cell lines (LCLs) in vitro. EBV can establish asymptomatic life-long persistence and is associated with multiple human malignancies, including non-Hodgkin lymphoma and Hodgkin lymphoma, as well as infectious mononucleosis. Although EBV-associated lymphomagenesis has been investigated for over 50 years, viral-mediated transformation is not completely understood, and the development of EBV-specific therapeutic strategies to treat the associated cancers is still a major challenge. However, the rapid development of several novel therapies offers exciting possibilities to target EBV-induced lymphomas. This review highlights targeted therapies with potential for treating EBV-associated lymphomas, including small molecule inhibitors, immunotherapy, cell therapy, preventative and therapeutic vaccines, and other potent approaches, which are novel strategies for controlling, preventing, and treating these viral-induced malignances.

## 1. Introduction

**Figure 1:**
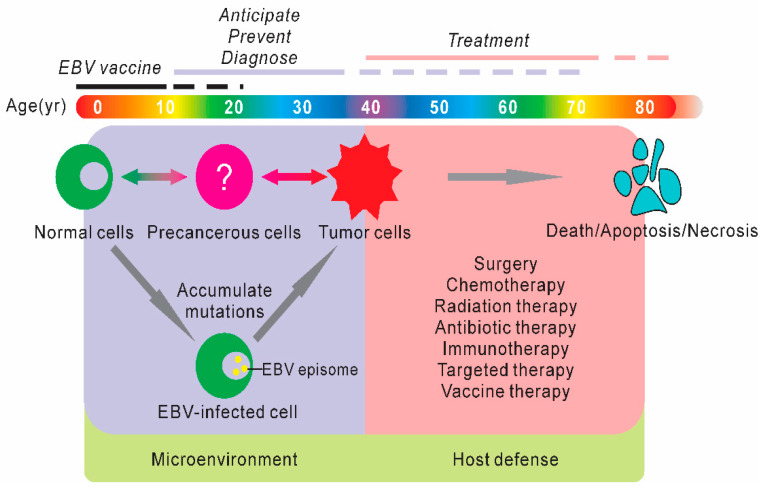
Schematic diagram of the representative protective or therapeutic strategies in development for Epstein-Barr virus (EBV)-associated diseases. EBV vaccination is always a high priority for children under 10 years old and there are no available commercial EBV vaccines. The morbidity and mortality of EBV-associated diseases are relatively low among young adults, so the first strategy is to anticipate, prevent, and diagnose the potential lymphomas. The majority of patients with EBV-associated lymphomas are more than 35 years old, and multiple approaches, including EBV-targeted therapies, are applied to treat these lymphomas. Although EBV is not able to induce the related lymphomas, it can play a critical role in the induction of a multistep process to carcinogenesis through accumulating mutations, in addition to other cofactors. These processes are highly associated with activities in the microenvironment and the host defense.

The global burden of cancer is a serious threat to public health due to the rapid growth in cancer incidence and mortality [1]. Approximately 2.2 million new cancer cases were associated with infectious agents in 2018 [2]. The estimated number of new cases of Hodgkin lymphoma and Burkitt lymphoma was about 92,000, 51% of which were attributable to Epstein-Barr virus (EBV) infection [2]. EBV was the first discovered human tumor virus that infects more than 90% of the human population worldwide. Notably, EBV establishes asymptomatic lifelong persistence and immortalizes human primary B-cells under specific conditions of immunocompetency. Latent infection is artificially defined based on the specific transcriptional profiles of viral-encoded antigens. In particular, the initial EBV infection of human naïve B-cells deploys a latency Ⅲ program with the characteristics of expressing all viral latent proteins, including EBV nuclear antigens (EBNA1, EBNA2, EBNA3A, EBNA3B, EBNA3C, EBNALP) and latent membrane proteins (LMP1, LMP2A, and LMP2B) [3]. The infected cells in this program are typically eliminated by cytotoxic T lymphocytes (CTLs), and then switched to a latency Ⅱ program with EBNA1, LMP1, and LMP2A expressed. The latency program can also switch to a latency Ⅰ program that expresses the EBNA1 antigen, or a latency 0 program only expressing EBV-encoded RNAs (EBERs). Importantly, the distinct latency programs are usually associated with specific lymphomas, and EBV latent antigens utilize multiple strategies to induce lymphomagenesis, although the detailed molecular mechanisms are still not fully known [3]. However, persistent EBV infection can serve as a perfect target for the treatment of EBV-associated lymphomas (Figure 1).

Targeting EBV-encoded proteins is the preferred strategy for treatments of EBV-associated lymphomas. During EBV latent infection, EBV expressed proteins are mostly immunogenic [4]. They can induce strong immune responses in immunocompetent individuals. Therefore, the majority of EBV latent antigens are suppressed under the pressure of a competent immune system, while they are expressed in immunodeficient hosts [5]. This elusive expression pattern restrains specific therapies by targeting these latent viral proteins in EBV-infected cells. In addition, targeting the EBV genome is another option for the targeted treatment of lymphomas, but the low copy numbers of viral DNA in these infected cells are restrictive towards the development of targeted therapies. In particular, EBV disturbs cellular homeostasis that can lead to lymphomagenesis. However, the virus itself may not be a necessary factor for lymphoma development and survival. Therefore, EBV-targeted therapies are only feasible for limited subtypes of lymphomas.

EBV-induced lymphomas show distinct hallmarks of cancer, such as resisting cell death, sustaining proliferative signaling, and evading growth suppressors [6]. Therefore, targeting EBV-associated signaling pathways linked to cancer is an efficient strategy for treating EBV-associated lymphomas. Previous studies have revealed that EBV latent genes are linked to the oncogenic activities in these specific lymphomas [3]. For example, LMP1 mimics the CD40 receptor-related signaling pathway to drive cell survival and growth [7,8]. LMP1 is also associated with three members of tumor necrosis receptor-associated factors (TRAFs) and mediates the activation of NF-κB in LMP1 transgenic mice, which may contribute to EBV-induced lymphomagenesis as survival signals [9,10]. LMP2A can constitutively induce Akt phosphorylation and activate the PI3 K/Akt signaling pathway that is dependent on phosphatidylinositol 3-kinase activity [11]. This may further inhibit TGF-β1 (transforming growth factor beta 1) mediated apoptosis and promote cell survival for lymphomagenesis [12]. Our recent study demonstrated that PI3Kγ is a potential target of Brusatol and its derived analogs in EBV-positive lymphomas [13]. Additionally, other important WNT and MAPK (mitogen-activated protein kinase) signaling pathways are also hijacked by EBV latent proteins to exhibit hallmarks of cancer during lymphomagenesis [14,15]. Therefore, these EBV-regulated cellular molecules or pathways are potential candidates for targeting lymphomas. Further investigations will reveal additional evidence as to clues that drive EBV-associated lymphomas and can be targeted by multiple treatments.

## 2. Discovery of Small Molecule Inhibitors

**Figure 2:**
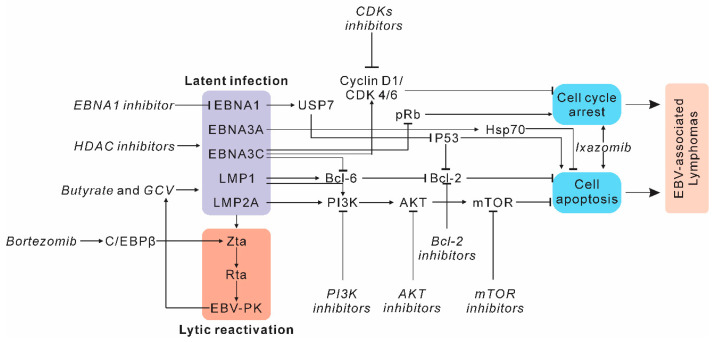
Therapeutic strategies for targeting EBV-associated lymphomagenesis. EBV-expressed latent proteins induce cell cycle arrest and cell apoptosis through the regulation of many crucial signaling pathways during lymphomagenesis. Specific small molecule inhibitors (italic font) that can target the key factors in these pathways are underlined.

EBV is a well-known oncovirus that can immortalize human B lymphocytes to lymphoblastoid cells. However, the detailed mechanisms by which viral antigens can modulate cellular transcription programs are not fully understood. Similar to other human oncoviruses, EBV is required, but not able, to induce oncogenesis [3,16]. Previous studies have shown that many host factors are hijacked during EBV infection and play key roles in EBV-induced lymphomagenesis (Figure 2). Therefore, these factors are potential targets for specific small molecule inhibitors.

### 2.1. Targeting Host Factors and Signaling Pathways in EBV-Induced Lymphomas

EBV-induced lymphomagenesis involves the dysregulation of numerous signaling pathways in infected cells, including B-cell receptor signaling, the PI3K (phosphoinositide 3-kinase) pathway, the JAK/STAT (Janus kinase/signal transducers and activators of transcription) pathway, the MAPK pathway, the NF-κB (Nuclear factor-κB) pathway, and others [15,17]. These molecules and signaling pathways are also potential targets for various therapeutic approaches in EBV-induced lymphomas. We present several important pathways and the associated targeted therapies below (Figure 2).

Cell cycle dysregulation is a hallmark of EBV-mediated oncogenesis [18]. Cyclin D and cyclin-dependent kinase (CDK) have been identified as potential therapeutic targets in cancers [19]. The EBV essential antigen EBNA3C facilitates G1/S transition and cell proliferation by stabilizing Cyclin D1 and Cyclin D2 proteins in EBV-transformed cells [20,21]. LMP1 can also induce Cyclin D2 expression to mediate uncontrolled cell proliferation [22]. Cyclin D1 overexpression is required for persistent EBV infection in nasopharyngeal epithelial cells [23]. Cyclin D/CDK activity is also regulated by the CDK inhibitory proteins, including the INK4 (Inhibitors of CDK4) family (INK4A, INK4B, INK4C, and INK4D) and the CIP/KIP (CDK interacting protein/Kinase inhibitory protein) family (p21, p27, and p57) [19,24,25]. EBNA3C also binds to Cyclin A and modulates its dependent kinase activity in EBV-infected cells [26,27]. EBNA3C can enhance Cyclin A/CDK2 kinase activity by suppressing p27-mediated inhibition [26]. Furthermore, the EBV immediate-early lytic transactivator Zta can induce CDK inhibitors and mediate G0/G1 cell cycle arrest to facilitate viral lytic replication. These studies have shown the critical roles of CDK inhibitors in the switch between latency and lytic infection [28]. Therefore, they are potential targets in the development of strategies for treating EBV-associated cancers.

Although EBV utilizes multiple mechanisms to block apoptosis and induce oncogenesis, targeting cell apoptosis is another viable therapeutic strategy for treating EBV-associated lymphomas. For example, targeting the antiapoptotic protein Bcl-2 is a promising strategy for treating lymphomas [29]. The EBV latent antigen LMP1 upregulates the oncogene Bcl-2 to protect EBV-infected cells from apoptosis, suggesting a key role for the survival of EBV-transformed cells [30]. Bcl-6 is a master regulator involved in germinal center (GC) B-cell development and is regulated by the viral latent antigen EBNA3C and cellular host IRF4 [31,32,33]. EBNA3C inhibits Bcl-6 activity and results in a derepression of Bcl-2 expression for lymphomagenesis [31]. ABT-199, which is a Bcl-2-selective inhibitor, can inhibit the growth of some tumors [34]. One study, however, showed that ABT-199 was ineffective for EBV-immortalized cells, but may have efficacy against early EBV-infected B-cells [35]. Further, the EBV-encoded Bcl-2 homolog, BHRFl, binds the Bcl-2-related protein Bim to block apoptosis [36]. An inhibitor specifically designed to target BHRF1 was shown to suppress tumor growth and extend survival in a mouse model of EBV-positive B-cell lymphoma [37].

The ubiquitin/proteasome signaling pathway is regulated by multiple EBV latent antigens during lymphomagenesis. EBNA1 can interact with the ubiquitin-specific protease HAUSP/USP7 and competitively binds to its cellular partners p53 and MDM2, which mediates p53 degradation and inhibits apoptosis [38,39]. Interestingly, the Gly-Ala repeat domain of EBNA1 may inhibit major histocompatibility complex (MHC) class I-mediated antigen processing via regulation of the ubiquitin/proteasome signaling pathway [40]. EBNA3C can regulate the ubiquitin-proteasome-dependent pathway to degrade or stabilize host factors (such as pRb, p53, and Bcl-6), in order to induce lymphomagenesis [31,41,42]. Ixazomib, which is an FDA-approved orally bioavailable proteasome inhibitor, is capable of inducing cell apoptosis and cell cycle arrest in EBV-positive lymphoma cells, suggesting its potential value in the treatment of EBV-associated lymphomas [43]. Another proteasome inhibitor called bortezomib induces CCAAT/enhancer-binding protein β (C/EBPβ) expression and activates EBV immediate-early Zta protein-mediated lytic infection in Burkitt lymphoma [44,45].

Additionally, spleen tyrosine kinase (SYK) was shown to be essential in the BCR (B-cell receptor) signaling pathway. A recent study demonstrated that a novel SYK inhibitor—TAK-659—inhibited tumor development by inducing cell death in EBV-associated lymphomas in vivo [46,47]. Although many host factors can play key roles during EBV-induced lymphomas, most of these potential candidates, including transcription factors, scaffolding, and regulatory proteins, are difficult to target using small molecular inhibitors. New emerging techniques provide notable strategies for the development of specific drugs as targeted therapies. The proteolysis targeting chimeras (PROTAC) technique can be employed to synthesize a bifunctional molecule that bridges a protein of interest (POI) and its specific E3 ligase, leading to degradation of the POI via the ubiquitin/proteasome signaling pathway [48,49]. The hydrophobic tagging (HyT) technique attaches a hydrophobic group to a small molecule targeting a POI. Binding of the bivalent molecule recruits endogenous chaperones and induces ubiquitin/proteasome-dependent degradation of the unfolded protein [48,50]. We have now introduced several EBV latent proteins that can interact with and degrade multiple important host factors. Therefore, these platforms may accelerate the discovery of new anti-EBV drugs for the treatment of EBV-related lymphomas.

### 2.2. Strategies of Specifically Targeting EBV Antigens in Lymphomas

EBNA1 is the only EBV latent antigen expressed in the different types of latency, making it a promising but challenging target in EBV latently-infected cells [51,52]. It is responsible for the attachment of the viral episome to human chromosomes and facilitates its segregation during cell division. The crystal structure of the DNA-binding domain of EBNA1 and the latent origin of replication (*oriP*) facilitate the rational design of EBNA1-specific inhibitors [53,54]. These initial EBNA1 targeting compounds can significantly inhibit the activity of wild-type EBNA1 [55]. The first EBNA1-specific inhibitors were identified using high-throughput computational docking programs with EBNA1, which were then employed to establish a biochemical high-throughput screening platform based on a homogeneous fluorescence polarization (FP) assay [51,52]. These experiments demonstrated the feasibility of identifying EBV-specific inhibitors with multiple platforms. The novel small molecule inhibitors that specifically block the DNA-binding domain of EBNA1 and inhibit tumor growth in vivo have also shown therapeutic potential [56]. A related clinical phase 1/2a trial is currently ongoing at Cullinan Apollo Corporation.

The EBV genome predominantly exists in infected cells as a viral episome and is intractable as a potential target for precision treatment. Hydroxyurea, which is a ribonucleotide reductase inhibitor, can eliminate EBV episomes from Burkitt lymphoma cells and EBV-immortalized lymphoblastoid cell lines [57]. A detailed mechanism for this has not yet been identified. Two patients with EBV-associated primary central nervous system lymphoma (PCNSL) were orally given low-dose hydroxyurea and survived from the normal duration of 4–6 weeks to over 21 months [58]. These experiments not only suggested the potential of hydroxyurea in the treatment of EBV-related PCNSL, but also showed the efficacy of eliminating EBV genomes from hosts as an anti-viral therapeutic strategy. However, specifically targeting the EBV genome may not be efficient enough to clear viral integration in the human genome. Further studies are needed to evaluate and develop this approach with additional clinical trials.

Another strategy for treating EBV-associated lymphomas is the induction of lytic infection in EBV-positive cells. The switch from latency to lytic replication is dependent on the expression of two immediate-early genes—*BZLF1* and *BRLF1* [59]. These two genes encode Zta and Rta proteins that activate a cascade of lytic genes and lead to the production of viral particles [59,60]. In EBV latently-infected cells, lytic reactivation can be achieved after treatment with various stimuli, including histone deacetylase (HDAC) inhibitors [61], 12-O-tetradecanoylphorbol-13-acetate (TPA) [62], sodium butyrate [63,64], and anti-immunoglobulin [65,66]. Five tetrahydrocarboline derivatives were identified through a high-throughput cell-based assay, and the most active compound C60 was effective at activating the EBV lytic cycle with less toxicity [67]. However, most of these treatments are not specific to EBV reactivation and also induce a number of strong side effects in patients. Therefore, these agents were used in combination with other therapies for EBV-induced lymphomas. For example, a clinical trial demonstrated that the combination of arginine butyrate and ganciclovir was an effective therapeutic approach against EBV-associated lymphomas [68]. Arginine butyrate induced the lytic cycle of EBV latently-infected cells and activated the *BXLF1*-encoded EBV thymidine kinase (EBV-TK), whose expression allowed EBV-infected cells to be susceptible to ganciclovir—a nucleoside-type antiviral agent that blocks viral replication [68,69]. However, another study demonstrated that *BGLF4*-encoded EBV protein kinase (EBV-PK), but not EBV-TK, was responsible for ganciclovir-mediated inhibition [70]. Further applications of these small molecule inhibitors may require a deeper understanding of their specific molecular mechanisms in induction of the EBV lytic cycle.

### 2.3. Application of CRISPR Therapeutics for the Treatment of EBV-Associated Lymphomas

EBV persists as an asymptomatic infection in host cells with low levels of viral episomes, which makes it difficult to eradicate the viral genome from infected cells. However, the development of the CRISPR/Cas9 system provides a possible strategy for the termination of latent infection. By introducing guide RNAs (gRNAs) that can target the regions of EBNA1 and *oriP*, the CRISPR/Cas9 system efficiently induced more than a 95% loss of EBV genomes [71]. This showed that the CRISPR/Cas9-mediated gene editing strategy may be a potent anti-viral therapy by targeting the critical regions of viral genomes in latent infection. The CRISPR/Cas9 screen has also been widely used to identify key factors that modulate viral latency or the lytic program and explore potential therapeutic targets for treating EBV-associated lymphomas. One CRISPR/Cas9 screen demonstrated that the ubiquitin ligase ubiquitin-like PHD and RING finger domain-containing protein 1 (UHRF1) and DNA methyltransferases (DNMT1 and DNMT3B) are necessary for the restriction of EBV latency Ⅲ-associated oncoproteins in Burkitt lymphoma [32]. Polycomb repressive complex I (PRC1)-mediated histone ubiquitylation also provides another mechanism of restricting viral latency [32]. These results further extend the rational and critical therapeutic targets that regulate viral protein expression. Another CRISPR/Cas9 screen showed that MYC was a major suppressor of the EBV lytic cycle in Burkitt lymphoma cells [72]. Although MYC was recognized as “undruggable” because of the serious side effects it induced in normal cells, the improved small-molecule MYC inhibitors still had potential for use as therapeutic agents [73,74]. Furthermore, the study demonstrated that the cohesin SMC1A, FACT, STAGA, and mediator can inhibit EBV reactivation by supporting MYC expression, and the depletion of MYC activated the EBV lytic cycle [72]. Furthermore, the FACT inhibitor CBL0137 significantly induced the expression of EBV lytic genes, suggesting that it may be a druggable target during the switch of latency and lytic replication [72,75].

## 3. Immunotherapy and Cell Therapy in EBV-Associated Lymphomas

Advances in immunotherapy have reshaped the lymphoma therapy landscape, and physicians have gained more flexibility to choose and design immunotherapeutic strategies, including immune checkpoint inhibitors, tumor-specific monoclonal antibodies, adoptive T-cell transfer, and cytokine immune system modulators. The first three options, through extensive development, have particularly improved treatment outcomes in EBV-associated post-transplant lymphoproliferative disorders (PTLD). We will provide an overview of the development and insights on possible further improvements in these strategies for the treatment of EBV-associated lymphomas.

### 3.1. Immune Checkpoint Inhibitors (PD-1/PD-L1 Antibody)

High PD-L1 expression is associated with a range of EBV-positive lymphomas, including post-transplant lymphoproliferative disorders (PTLD), diffuse large B-cell lymphoma (DLBCL), Burkitt lymphoma (BL), plasmablastic lymphoma (PBL), and natural-killer/T-cell lymphoma (NKTCL) [76,77,78,79,80]. EBV-transformed lymphoblastoid cells also express high levels of surface PD-L1, and the EBV latent membrane protein LMP1 enhances PD-L1 promoter activity through JAK/STAT signaling [78]. LMP1 was also found to be responsible for the upregulation of PD-L1 expression through NF-κB activation in NKTCL [77]. EBNA2 is responsible for PD-L1 upregulation by downregulating the miR-34a repressor in DLBCL [76]. Moreover, it has been suggested that the tumor microenvironment in EBV-associated lymphomas is rich in PD-1/PD-L1 active engagements, and PD-1 expressing tumor-infiltrating lymphocytes are associated more with EBV-positive tumors [78,80]. A subset of BL samples are characterized by M2-macrophage polarization, activated PD-L1 expression, and non-canonical LMP2A expression in the tumor microenvironment [81]. EBV-positive DLBCL cells exhausted T-cells and induced their PD-1 expression in vitro; however, introducing a PD-1 blockade can restore T-cell activity [82]. The above-mentioned results suggest that the PD-1/PD-L1 signaling pathway is a viable target in relieving immune checkpoint evasion. 

In a clinical trial conducted in patients with relapsed or refractory non-Hodgkin lymphomas, all patients with EBV-negative tumors failed to respond to the anti-PD1 antibody pembrolizumab (0/15), while seven patients out of 15 with EBV-positive tumors responded to the treatment (7/15, 47%) [83]. The overall response rates in NKTCL (6/14, 44%) and primary mediastinal B-cell lymphoma (PBMCL) (1/4, 25%) were higher than in EBV-negative DLBCL (0/10) and T-LBL (0/2). None of the patients showed any grade 3 or 4 pembrolizumab-associated toxicities. In another clinical trial on treating NKTCL after failing L-asparaginase (L-ASP), all patients responded to pembrolizumab (7/7) [84]. Five patients achieved a complete response (5/7, 71%), with two of them having undetectable EBV DNA. Three out of four patients with high PD-L1 expression achieved a complete response, while the patient with low PD-L1 expression achieved a partial response. No treatment-related adverse events were observed during the trial. These two clinical trials showed that a PD-1 blockade with pembrolizumab could be a viable option when treating relapsed or refractory EBV-positive NKTCL. Although no statistical analysis showed that high PD-L1 predicts a better treatment outcome, the manageable toxicity observed in these two clinical trials demonstrated that the PD-1 blockade could be considered as a salvage therapy strategy in other high PD-L1 expressing EBV-associated lymphomas. Several ongoing clinical trials combining the PD-1/PD-L1 pathway and targeting other critical viral or cellular factors have provided new regimes that could augment the response concurrently with anti-PD-1 treatment (Table 1).

Introducing a blockade in both PD-1 and CTLA-4 may also increase the tumor-targeting potency. In a humanized mouse model, the effect of growth inhibition on EBV-induced lymphomas was greater in a dual blockade than treatment with anti-PD-1 or anti-CTLA-4 alone [85]. A PD-1/CTLA-4 blockade also increased EBV-specific T-cell responses, leading to a decrease in the overall number of EBV-positive B-cells [85]. The efficiency and safety of a PD-1/CTLA-4 dual blockade are currently being investigated in other types of cancer. However, further adjustments in the overall regimen would have to address the seemingly higher toxicity observed in combination therapies [86]. 

The microRNA miR-155, which is more abundant in EBV-positive DLBCL cells, could be used as a potential prognostic indicator for the effectiveness of anti-PD-L1 treatment [87]. miR-155 modulates the PD-1/PD-L1 interaction by upregulating PD-L1 in DLBCL cells, but cells with higher miR-155 levels are in turn more sensitive to a PD-L1 blockade. Serum miR-155 levels also positively correlated with miR-155 levels in tumors [87].

### 3.2. Monoclonal Antibodies

The anti-CD20 antibody rituximab is currently being used to treat PTLD by inducing B-cell depletion, and the reported overall response rate to anti-CD20 treatment alone ranges from 32% to 79% in PTLD patients [88]. Rituximab is also used in combination with cyclophosphamide, doxorubicin, vincristine, and prednisone (CHOP) in EBV-positive DLBCL and PTLD [88,89]. Overall, rituximab-related toxicity has been reported to be very manageable. Recent studies have discussed the possibility of using rituximab as a prophylaxis approach for patients either pre-transplant or post-transplant with suspected EBV-reactivation detected in peripheral blood [90,91,92]. The overall incidence of PTLD reported after pre-transplant rituximab was reduced, and most patients who received preemptive rituximab post-transplant did not develop PTLD [90,91,92]. 

Although anti-CD20 has been proven to be effective in EBV-positive lymphomas, it does not specifically target EBV-positive cells as CD20 is expressed on most normal B-cells. Notably, it was reported that CD70, which is only expressed in highly activated B- or T-cells, could be an alternative target in EBV-associated lymphomas [93].

### 3.3. Adoptive EBV-Specific T-Cell (EBVST) Therapy

Adoptive T-cell therapy aims to restore immunity in patients with post-transplant immunosuppression, in which EBV reactivation poses a great risk towards EBV-associated diseases. The infusion of unseparated donor leukocytes showed promising results in treating PTLD; however, risks that arose from the graft-verse-host disease could not be ignored [94]. To provide better safety, EBV-specific T-cell therapy was developed to provide specific recognition to EBV antigen-presenting B-cells. In particular, donor leukocytes were extracted, stimulated by antigen-presenting cells such as lymphoblastoid cell lines (LCLs), further expanded, and injected back into the patient. In a retrospective study spanning more than 10 years on 114 patients who underwent EBVST infusion, all patients who received prophylactic infusions did not develop PTLD, 80% of existing PTLD patients had a complete response, and the overall toxicity was minimal [95,96,97]. Later trials conducted with EBNA-1 or LMP-specific CTLs also showed encouraging results on PTLD or EBV-positive Hodgkin and non-Hodgkin lymphoma, respectively [98,99]. 

Although EBVST treatment resulted in a satisfactory response rate with low toxicity, it was mostly limited by a lengthy preparation time and low readiness. Allogenic EBVST banks prepared from healthy donors, or third party “off-the-shelf” products, are currently being developed to solve this issue [100,101]. Table 1 also shows the representative ongoing clinical trials. 

### 3.4. T-Cell Receptor-Modified T-Cell Therapy

Engineering T-cells to express EBV antigen-specific T-cell receptors (TCR) is an alternative strategy for producing potent cell therapy in a relatively short timeframe. This approach also provides flexibility in epitope designs, thus allowing easier optimization inefficiency of the final product.

One group attempted to generate modified TCRs that are specific to either the EBNA3A, EBNA3B, or BamHI-M leftward reading frame [102]. Neither cytotoxicity assays nor cytokine (IFN-γ or TNF-α) production were detected in TCR-modified T-cells upon contacting native LCLs [102]. Introducing a CD28 domain into the modified design improved IFN-γ production upon the reaction. However, the modified T-cells remained unresponsive. The suboptimal TCR design and cell handling may be responsible for the unresponsiveness. Alternatively, attempts to develop late membrane protein-specific modified-TCR seemed to be more successful; LMP1- and LMP2A-specific TCR conferred a T-cell response in mouse lymphoma models [103,104,105]. Seemingly, the choice of epitope could be crucial in modified-TCR design.

Furthermore, TCR promoters could be a point for optimization. The TCR-specific promoter Vβ6.7 produces a superior effect on LMP2A-specific TCR expression and downstream LMP2A-specific T-cell activity [105]. Adjusting the promoter strength could be a potential strategy for optimizing the overall therapeutic potency.

### 3.5. Chimeric Antigen Receptor T-Cell Therapy

Chimeric antigen receptor (CAR)-modified T-cells are designed to overcome the tolerance in conventional adoptive therapy to tumors that lack MHC antigen presentation [106]. Since current CARs are constructed with an extracellular antigen-specific scFv (single-chain Fv) and intracellular co-stimulatory domains, CAR T-cells could engage their designated specific cell surface antigen and elicit cytotoxic response, independent of MHC presentation [107,108]. Currently, CD19-specific CAR T-cells are the best-studied for treating B-cell malignancies [107]. CD19 is an excellent target as it is only present on B-cells. Although the effects of CD19 CAR T-cells have been impressive, this treatment is also associated with toxicities related to off-target B-cell death, such as cytokine release syndrome, encephalopathy, and B-cell aplasia. Increasing the specificity to tumor cells may help to reduce B-cell cytotoxicity [108].

Donor EBV-specific T-cells were modified to express CD19 CAR and were used in a phase 1 clinical trial to treat relapsed B-cell malignancies after an allogeneic stem cell transplant [109]. Two out of six patients responded to the treatment, and two patients received treatment while remaining in remission [109]. Three patients experienced EBV viral reactivation, and CD19 CAR-modified virus-specific T cells (CD19 CAR VST) in two of them seemed to expand due to this viral stimulation. Allogenic CAR T therapy could be a more economical approach towards EBV-associated lymphomas. Allo-EBV.CD19.CAR T-cells are currently under development in vitro, having displayed cytotoxicity towards CD19-positive EBV-positive cells and little reactivity towards HLA (human leukocyte antigen) mismatched cells [110]. Further research will help to provide other off-the-shelf options for patients with PTLD.

Another potential CAR target in EBV-associated lymphoma is latent membrane proteins, which are present on the cell surface during latency program II and III. This has been proven to be a potential approach against nasopharyngeal carcinoma in vitro and in a mouse model, as an infusion of LMP1 HELA/CAR T-cells inhibited NPC tumor growth in a xenograft model [111]. This approach would also be viable in LMP1-positive lymphomas and needs further investigation.

## 4. Preventative and Therapeutic Vaccines for EBV Infection

There is still no commercially available EBV vaccine against EBV-associated lymphomas. The EBV glycoprotein gp340 was initially used as a prototype subunit vaccine that offered protection against EBV infection or EBV-induced lymphomas in cottontop tamarins, but the gp340-specific antibody was unable to neutralize EBV in vitro [112,113,114]. Subsequently, the EBV glycoprotein gp350 was shown to be the principal antigen for inducing neutralizing antibodies against B-cell infection in human sera [115,116]. EBV gp350 mediates B-cell infection through binding to the complement receptor 2 (CR2/CD21) and represents a promising target for neutralizing antibodies [116,117]. A recombinant gp350 protein from Chinese hamster ovary (CHO) cells elicited high neutralizing EBV-specific antibody titers in rabbits, suggesting that it was a potential candidate for a subunit vaccine against EBV-associated cancers [118]. By studying the binding site of gp350 and CR2/CD21, a potent epitope was structurally designed as a nanoparticle vaccine that induced neutralizing antibodies in mice and non-human primates [119]. This provides a new strategy that focuses on a conserved viral domain for the design of an EBV vaccine. However, a phase Ⅱ clinical trial with a recombinant EBV subunit glycoprotein 350 (gp350)/aluminum hydroxide and 3-O-desacyl-4′-monophosphoryl lipid A (AS04) candidate vaccine demonstrated that this vaccine failed to prevent asymptomatic EBV infection, despite showing safety and reactogenicity [120,121]. These results suggest that gp350-specific antibodies may reduce the risk of EBV-associated disease, but are not able to prevent EBV infection in humans. Of particular importance is that the CR2-binding site of gp350 was remarkably conserved, but the gp350 gene was diverse, in patients with primary EBV infection [122]. This observation provides new insight into the development of future EBV vaccines by targeting the viral gp350 protein.

To improve the immunogenicity of monomeric subunit vaccines, a feasible approach is to utilize multimeric proteins against EBV infection or EBV-associated lymphomas. Besides the gp350 protein, the glycoproteins gH/gL and gp42 were the majority of components that generated neutralizing antibodies which prevented B-cell infection [123]. The newly developed gH/gL and gH/gL/gp42 nanoparticle vaccines elicited neutralizing antibodies in mice and non-human primates and contributed to the neutralization of B-cell infection [123]. Antibodies induced by these nanoparticle vaccines inhibit EBV glycoproteins-mediated cell membrane fusion through targeting a site on gH/gL [123]. This approach may be a promising strategy for developing an effective EBV vaccine. Furthermore, an antibody against gH/gL—AMMO1—was isolated from rare memory B-cells and shown to neutralize EBV infection [124]. The CryoEM structure of the gH/gL-gp42-AMMO1 complex demonstrated that AMMO1 inhibited the EBV fusion machinery through a discontinuous epitope, which may be a potential target for the design of novel vaccines to block EBV infection [124]. However, a recent study described that traces of EBV infection could be detected in relapsed EBV-negative Hodgkin and non-Hodgkin lymphomas, suggesting the complicacy of developing specific prophylactic vaccines against EBV-associated lymphomas [125].

Virus-like particles (VLPs) are structurally similar to the parent virus, but devoid of the viral genome. They were considered as a potential vaccination candidate because of their superior safety and efficacy. An available approach of producing EBV VLPs is to use an engineered HEK293 cell line that lacks the terminal repeats (TR) responsible for DNA packaging and several EBV proteins (EBNA2, EBNA2A, EBNA3B, EBNA3C, LMP1, and BZLF1), but these cells have been shown to express the viral proteins essential for assembly and release [126,127,128]. The DNA-free VLPs were able to be captured by human B-cells and elicited high-titer neutralizing antibodies and a strong immune response in a murine model [126,127,128]. Another alternative strategy for producing EBV VLPs is to delete BFLF1/BFRF1A and gB (glycoprotein B) from the EBV genome, which may increase the purity and safety of DNA-free VLPs [129]. EBV VLPs were able to contain more viral latent antigens by fusing to the major tegument protein BNRF1, and stimulated cytolytic CD4^+^ T-cells against EBV infection [130]. The development of EBV VLPs is another promising approach to EBV vaccination; however, we are not certain whether it is practical or acceptable to use in humans.

Therapeutic vaccines are designed to boost cellular immunity in patients with EBV-associated diseases. A multicenter phase I clinical trial investigated the safety and immunogenicity of a therapeutic vaccine—MVA-EL—which encodes the C-terminal domain of EBNA1 and full-length LMP2 fusion proteins [131,132]. MVA-EL simultaneously reactivated EBNA1-specific CD4^+^ T-cell responses and LMP2-specific CD8^+^ T-cell responses in the context of nasopharyngeal carcinoma [133]. This clinical trial in Hong Kong was conducted in patients with nasopharyngeal carcinoma and it determined that all patients were in remission for more than 12 weeks [131]. The vaccination elicited a strong immune response to one or both viral antigens (EBNA1 or/and LMP2), without resulting in dose-limiting toxicity [131]. The following clinical trial in the United Kingdom (UK) performed in EBV-positive nasopharyngeal carcinoma patients also showed an increased T-cell response in eight of 14 patients [132]. EBV DNA was detected in four patients before vaccination, but two of them had an increased EBV load after vaccination [132]. These studies demonstrated that this EBV-specific recombinant vaccinia vaccine (MVA-EL) was safe and immunogenic for the treatment of EBV-associated cancers. Further large-scale trials are needed to determine its safety and efficacy. Although only EBV-associated nasopharyngeal carcinomas were tested in these mentioned clinical trials, this therapeutic vaccine specifically targeting viral EBNA1 and LMP2 antigens has the potential to be widely used against other EBV-positive lymphomas. Moreover, inducing T-cell responses may be a potential strategy for the development of EBV-specific therapeutic vaccines, although they are currently used for treatment. For example, Epstein-Barr virus (EBV)-specific cytotoxic T lymphocytes (CTLs) were successfully generated from nine Hodgkin lymphoma patients with active disease or in remission, and infused into three patients to maintain the antiviral activities for more than 13 weeks [134]. This demonstrated the feasibility of producing EBV-specific CTLs to treat EBV-associated Hodgkin lymphomas. LMP1 and LMP2 are also the primary targets in Hodgkin or non-Hodgkin lymphoma exhibiting the EBV latency Ⅱ program [135,136]. Autologous LMP-cytotoxic T lymphocytes (CTLs) that target LMP2 or LMP1 and LMP2 antigens were expanded and infused into 50 patients with EBV-associated lymphomas. The results showed that these LMP-specific CTLs achieved durable remissions without significant toxicity in 28 high-risk or multiple-relapse patients [98].

mRNA vaccines also represent promising approaches, with the characteristics of a high potency, rapid development, and low cost, but their wide application should overcome the restrictions of instability and inefficiency in vivo [137]. As far as the EBV vaccine is concerned, a developing mRNA vaccine encoding five EBV glycoproteins (gp350, gH/gL/gp42, and gB) by Moderna Therapeutics may reduce the rate of EBV-associated infectious mononucleosis (IM) and possibly prevent EBV infection. This mRNA vaccine is in preclinical development, so the efficacy still needs further investigation. Additionally, molecular Clamp is a new technology that generates chimeric polypeptides mimicking the pre-fusion structures of viral fusion proteins, and can rapidly develop anti-fusion vaccines or inhibitors against the enveloped viruses [138]. This novel method has already been used to develop specific vaccines targeting influenza, HIV, Ebola virus, and SARS-CoV-2.

## 5. Conclusions

Generally, targeted therapies for EBV-associated lymphomas aim to inhibit the critical signaling pathways for EBV-induced survival in lymphoma cells, activate the viral lytic cycle that is susceptible to antiviral therapies, or boost the immune response in patients with EBV-induced lymphomas. Although numerous cellular signaling pathways are modulated by EBV proteins in lymphomagenesis, most of these signaling pathways are also necessary for a normal cell life cycle. Targeting these pathways or molecules inevitably introduces moderate or serious side effects, so safety is a high priority in these treatments. More importantly, the lack of EBV-specific inhibitors is still a major bottleneck of anti-viral therapies. For example, the R-CHOP (rituximab, cyclophosphamide, doxorubicin, vincristine, and prednisone) commonly used for the treatment of non-Hodgkin lymphomas is not related to EBV infection, despite the fact that it is currently used to treat EBV-associated lymphomas [89,139]. Furthermore, the inducers of EBV lytic reactivation are mostly too toxic to be used in clinical trials. HDAC inhibitors are the most promising drugs that are being tested in clinical trials [140,141,142]. The treatment of EBV-associated cancers may benefit from these trials, but there are still no EBV-specific therapies. Emerging clinical drugs and drug-discovery platforms can provide new insights into the development of anti-EBV therapies, especially for exploring the specific inhibitors that can regulate EBV latency or lytic replication. Promising immunotherapy and cell therapy will continue to lead novel treatments for some types of EBV-associated lymphomas. Understanding the path of EBV-induced immortalization still requires much work, even with the support of diverse disciplines, but these findings will accelerate the development of targeted therapies for EBV-mediated lymphomas.

## Figures and Tables

**Table 1:** Representative active clinical trials on treating EBV-associated lymphomas.

Identifier	Year Started	Study Title	Phase
PD-1/PD-L1			
NCT04058470	2019	Toripalimab in Combination with R-CHOP for Elderly Patients with Untreated Diffused B-Cell Lymphoma	I/II
NCT04181489	2019	Sintilimab in Combination with R-CHOP in Patients with Treatment-naive EBV-positive Diffuse Large B-cell Lymphoma (DLBCL), NOS	II
NCT04084626	2019	PD1 Antibody and Lenalidomide as a Treatment for EBV-associated Hemophagocytic Lymphohistiocytosis (HLH) or Chronic Active EBV Infection (CAEBV)	III
NCT03586024	2018	Pembrolizumab in Relapsed/Refractory Extranodal NK/T- Cell Lymphoma, Nasal Type and EBV-associated DLBCL	I/II
NCT03258567	2017	Nivolumab in EBV-Positive Lymphoproliferative Disorders and EBV-Positive Non-Hodgkin Lymphomas	II
NCT03038672	2017	Nivolumab with or without Varlilumab in Treating Patients with Relapsed/Refractory Aggressive B-cell Lymphomas	II
**T-cell Therapy**			
NCT04156217	2019	EBV-TCR-T Cells for EBV Infection and EBV-Associated Post-Transplant Lymphoproliferative Disease After HSCT	I
NCT03789617	2018	Evaluate the Efficacy and Safety of EBV Induced Natural T Lymphocyte (EBViNT) Cell in Patients with Progressive EBV Positive Extranodal NK/T-cell Lymphoma Where Standard Treatments Have Failed	I/II
NCT03671850	2018	VT-EBV-N for Treatment of Severe in EBV Positive Extranodal NK/T Cell Lymphoma Patients	II
NCT03394365	2018	Tabelecleucel for Solid Organ or Allogeneic Hematopoietic Cell Transplant Participants with EBV-Associated Post-Transplant Lymphoproliferative Disease (EBV+ PTLD) After Failure of Rituximab or Rituximab and Chemotherapy	III
NCT03392142	2018	Tabelecleucel for Allogeneic Hematopoietic Cell Transplant Subjects with EBV-Associated Post-Transplant Lymphoproliferative Disease (EBV + PTLD) After Failure of Rituximab (MATCH)	III
NCT03044743	2017	PD-1 Knockout EBV-CTLs for Advanced Stage EBV Associated Malignancies	I/II
**Modified-TCR**			
NCT01956084	2013	Cytotoxic T Cells to Treat Relapsed EBV-positive Lymphoma (ALCI2)	I
**CAR-T Therapy**			
NCT03233854	2017	CD19/CD22 Chimeric Antigen Receptor (CAR) T Cells in Adults with Recurrent/Refractory B-Cell Malignancies	I
**HDAC Inhibitors**			
NCT03397706	2018	Dose Escalation & Expansion Study of Oral VRx-3996 & Valganciclovir in Subjects with Relapsed/Refractory EBV-Associated Lymphoid Malignancies	I/II
**Monoclonal Antibodies**			
NCT02924402	2016	Study to Evaluate Safety and Tolerability of XmAb13676 in Patients with CD20-expressing Hematologic Malignancies	I
NCT02670616	2016	Study of Ibrutinib in Combination with Rituximab-CHOP in EBV-positive Diffuse Large B-cell Lymphoma	II
